# Synthetic slings in the treatment of urinary incontinence: lessons learned and future perspectives

**DOI:** 10.1590/S1677-5538.IBJU.2020.04.04

**Published:** 2020-05-20

**Authors:** Cássio L. Z. Riccetto

**Affiliations:** 1 Universidade Estadual de Campinas CampinasSP Brasil Professor Livre Docente em Urologia, Universidade Estadual de Campinas – UNICAMP, Campinas, SP, Brasil;; 2 Hospital de Clínicas Faculdade de Ciências Médicas Universidade Estadual de Campinas CampinasSP Brasil Divisão de Urologia Feminina - Hospital de Clínicas da Faculdade de Ciências Médicas da Universidade Estadual de Campinas – UNICAMP, Campinas, SP, Brasil

## COMMENT

In the past decades, the refinement of therapeutic solutions for stress urinary incontinence and pelvic organ prolapse have evolved almost in parallel. Although initial proposals for the use of synthetic suburethral slings date back to the 1990s ( 1 ), their use became widespread in the 1990s, notably through the development of monofilament polypropylene slings. After the publication of the Integral Theory ( 2 ) and its materialized surgical application, the Tension-Free Vaginal Tape (TVT) quickly became the new gold-standard treatment of stress urinary incontinence in women. Thereafter, retropubic mid-urethral slings evolved, through the novel use of the transobturator approach ( 3 ) in order to achieve similar effectiveness with lower rates of surgical complications. Lastly, minislings ( 4 ) stemmed from the effort to make the procedure even less invasive, however its possible indications and long-term effectiveness still demand further investigation ( 5 , 6 ). Regardless of the approach taken by each technique, it is clear that the large amount of high-quality trials on mid-urethral slings have set them among some of the most well studied procedures in contemporary Urology.

After the development of the first mid-urethral mesh slings, a will to use mesh for the repair of pelvic organ prolapses did not take long to follow. From the start of the 2000s onwards, there was a significant increase in de number of procedures using polypropylene prostheses, which obtained prompt approval from international regulatory agencies based on the principle of material equivalence with mid-urethral synthetic slings.

Stress urinary incontinence and pelvic organ prolapse do indeed share similar risk factors and are akin to each other in their pathophysiology, based on progressive degeneration of collagen fibers in pelvic floor tissues, notably their supporting conjunctive fascia. Both conditions tend to become more prevalent with continuing increases in life expectancy, therefore representing a potential public health challenge in the near future, even in countries with good standards of perinatal care. Be that as it may, when compared to those with stress incontinence, patients with pelvic organ prolapse present more complex anatomical changes and a myriad of clinical presentations, in which voiding, proctologic or sexual dysfunction symptoms may predominate, depending on the mostly affected vaginal compartment (anterior, apical or posterior).

Due to such diversity in clinical presentation, initial attempts to standardize and promote the treatment of pelvic organ prolapse using transvaginal meshes proved to be inadequate, unlike their mid-urethral sling counterparts years ago. As most mesh kits were unable to repair combined vaginal wall defects, large or combined prostheses were required, leading to the need to implant large amounts of synthetic material in the vagina. As the vaginal elasticity is the main determinant of its normal physiology, the implant of inextensible material to treat a prolapse could lead to a significant risk of complications, such as voiding dysfunction, chronic pain and sexual dysfunction resulted from the local fibroblastic reaction, which can assume a permanent and progressive pattern. Thus, the increased implantation of transvaginal prosthesis for the treatment of pelvic organ prolapse was followed by a marked rise in the frequency of such complications, alongside vaginal exposure or extrusion of prosthetic segments and erosion of surrounding pelvic viscera.

The initial reaction to this evidence came from the Food and Drug Administration (FDA) through the publication of alerts in 2009 and 2011 suggesting caution in the use of transvaginal meshes ( 7 ), and another in 2012, which ordered mandatory prospective studies conducted by the companies who shared that market (2012) ( 8 ). Such warnings triggered the reaction of the lay community, initially in the United States, directed indistinctly against transvaginal meshes for POP as well as against synthetic mid-urethral slings, which included sensationalist reports published in the media, government inquiries and a significant increase in lawsuits against doctors and mesh manufacturer companies. The decision of Johnson & Johnson, one of the most relevant companies in the field, to withdraw from female pelvic medicine market in 2012, had great repercussions in North America and included not only prostheses for POP treatment, but also their mid-urethral sling brands.

On the other hand, international medical societies, such as the International Continence Society, International Urogynecological Association and Society of Urodynamics, Female Pelvic Medicine & Urogenital Reconstruction, released statements in order to uphold the great advance that synthetic medium urethral slings posed in treatment of stress urinary incontinence, and also to determine scientific criteria for the use of transvaginal mesh in the treatment of pelvic organ prolapse, recommending its use specifically for recurrences and stage 3 and 4 prolapses, especially vaginal vault prolapses ( 9 ). In fact, since 2008, the FDA has also systematically differentiated the transvaginal mesh for POP from synthetic mid-urethral slings and excluded special warnings against synthetic slings from its recommendations in 2011. However, in 2016 the FDA reclassified transvaginal prostheses for POP from category 2 to category 3, in a category akin to other implants such as heart valves, pacemakers, cochlear implants and intraocular lenses. Mesh manufacturers also made an effort towards reducing the amount of synthetic material implanted in pelvic organ prolapse surgeries, through low weight meshes and the refinement of anchoring systems, which changed from transobturator and transgluteal fixation, used in the early transvaginal prostheses, to sacrospinal ligament fixation devices, intending to prevent the risk of muscle bleeding, nerve compression, severe chronic pain and sexual dysfunction. Despite the technical improvements, campaigns against transvaginal meshes became popular in the United Kingdom, Australia and New Zealand, which culminated in an almost complete abolishment of the use of transvaginal meshes and synthetic slings in those countries, increasing the animosity to polypropylene prostheses through the world.

Reluctance in the use of transvaginal mesh for pelvic organ prolapse has led to its replacement by sacral colpopexy/hysteropexy, mostly driven by developed countries due to an increased availability of robot-assisted laparoscopy, which shortened the conventional laparoscopy learning curve. Thus, sacral colpopexy/hysteropexy quickly came to be the new standard technique for the treatment of pelvic organ prolapses, particularly vaginal vault prolapses, despite scientific references still indicating the need for further studies ( 10 - 12 ). In comparison, the experience with the former transvaginal meshes for POP necessarily leads to reflections about the future consequences of laparoscopically or robotically implanted meshes on vaginal elasticity and on the function of the pelvic floor, as some groups advised its fixation on the fascia of the levator ani muscle.

The aversion to synthetic slings rekindled the interest in fascial sling ( 13 - 15 ), which had been reserved mostly for complex cases or when incontinence was associated with specific conditions, such as urethral diverticula. Unlike in the 1990s literature, when evidence on aponeurotic slings was almost entirely based on case series with short or intermediate follow-up and a few unicentric prospective studies using homemade slings, nowadays the aponeurotic slings are being faced against commercially synthetic mid-urethral slings, using internationally validated and standardized objective and subjective healing criteria, applied in prospective multicenter randomized studies sometimes grouped by means of systematic review and meta-analysis techniques ( 16 ).

This ongoing trend has already provided the literature with evidence that tends to consider that the objective and subjective cure rates of synthetic and aponeurotic slings are similar, although aponeurotic slings have higher costs and more frequent adverse effects, even considering that modern aponeurotic slings became less wide and implanted in the urethra ( 17 ) instead in the bladder neck as originally proposed ( 18 ). From a qualitative point of view, recent research pointed out that nowadays aponeurotic slings are mainly performed by urologists, who are used to treating older patients with more comorbidities ( 19 ), and the risk of adverse events is directly related to surgical volume, being significantly lower for those surgeons who operated more than 50 cases per year ( 20 ).

In Brazil, synthetic medium urethral slings and some transvaginal meshes are still approved by the National Health Surveillance Agency and have been used by urologists and gynecologists based on their own judgment. Moreover, the fact that these treatments are barely offered by the Public Health System, and the higher age of the majority of patients with pelvic organ prolapse who seek medical assistance – most without great sexual expectations – could contribute to alleviate part of the eventual dissatisfaction generated by possible adverse effects. Nevertheless, it is highly recommended that a signed informed consent form is obtained, and the surgeon should maintain a prolonged post-operative follow-up in order to detect and treat adverse events as early as possible.

We must recognize that expectations regarding the treatment of stress urinary incontinence tend to irreversibly increase over the next years, since they are significantly enclosed in female quality of life. Moreover, a growing number of elderly women tend to practice physical activities regularly and remain sexually active for longer periods. Also, despite all efforts, research on how to modulate pelvic floor collagen degradation and remodeling through genetic engineering has not yielded any significant results yet, leaving surgical treatments as still the main therapeutic alternative for incontinence in the near future.

In conclusion, the proper use the synthetic mid-urethral slings and meshes for pelvic organ prolapse is now a choice of the pelvic floor reconstructive surgeon, should prioritize their use in ligament reinforcement rather than fascial replacement, while also committing to monitoring patients more carefully for longer. Simplistic solutions, such as the indistinct ban of promising technologies, or even a massive reinvigoration of old techniques previously replaced due to their adverse effects or their dissonance to modern pathophysiological concepts about incontinence, certainly do not represent the best alternative for the care of our patients.
